# The Evaluation of Preference and Perceived Quality of Health Communication Icons Associated with COVID-19 Prevention Measures

**DOI:** 10.3390/healthcare9091115

**Published:** 2021-08-27

**Authors:** Yogi Tri Prasetyo, Ratna Sari Dewi, Naiomi M. Balatbat, Michael Lancelot B. Antonio, Thanatorn Chuenyindee, Anak Agung Ngurah Perwira Redi, Michael Nayat Young, John Francis T. Diaz, Yoshiki B. Kurata

**Affiliations:** 1 School of Industrial Engineering and Engineering Management, Mapúa University, 658 Muralla St., Intramuros, Manila 1002, Philippines; yumibalatbat@yahoo.com (N.M.B.); michaellancelotantonio@gmail.com (M.L.B.A.); earthclubz@windowslive.com (T.C.); mnyoung@mapua.edu.ph (M.N.Y.); ybkurata@ust.edu.ph (Y.B.K.); 2 Department of Industrial and Systems Engineering, Kampus ITS Sukolilo, Institut Teknologi Sepuluh Nopember, Surabaya 60111, Indonesia; ratna@ie.its.ac.id; 3 School of Graduate Studies, Mapua University, 658 Muralla St., Intramuros, Manila 1002, Philippines; 4 Navaminda Kasatriyadhiraj Royal Thai Air Force Academy, Bangkok 10220, Thailand; 5 Industrial Engineering Department, BINUS Graduate Program—Master of Industrial Engineering, Bina Nusantara University, Jakarta 11480, Indonesia; wira.redi@binus.edu; 6 Department of Finance and Accounting, Asian Institute of Management, 123 Paseo de Roxas, Legazpi Village, Makati 1229, Philippines; jdiaz@aim.edu; 7 Department of Industrial Engineering, Faculty of Engineering, University of Santo Tomas, España Blvd, Manila 1015, Philippines

**Keywords:** COVID-19, medical icon, ranking test, perceived quality, semantic distance, usability

## Abstract

Icons have been widely utilized to describe and promote COVID-19 prevention measures. The purpose of this study was to analyze the preference and subjective design features of 133 existing icons associated with COVID-19 prevention measures published by the health and medical organizations of different countries. The 133 icons represent nineteen different function names, such as “Wash Hands” and “Wear Face Mask”. A total of 57 participants were recruited to perform two different tests: ranking test and subjective rating test. The ranking test was conducted to elicit the preference ranking of seven icon designs representing each function name. It was followed by a subjective rating test using 13 semantic scales on the two most preferred icons to analyze their perceived quality. Spearmen correlation was applied to derive the possible correlations between users’ rankings and the semantic scales, and Friedman’s test was also performed to determine the true difference between ranking in terms of each semantic scale to provide a fully meaningful interpretation of the data. Generally, findings from the current study showed that the image presented in the icon is the key point that affects the icons’ perceived quality. Interestingly, Spearman’s correlation analysis between preference ranking and semantic scales showed that vague–clear, weak–strong, incompatible–compatible, and ineffective–effective were the four strongest semantic scales that highly correlated with the preference ranking. Considering the significant relationships between the semantic distances and the functions, images depicted in an icon should be realistic and as close as possible to its respected function to cater to users’ preferences. In addition, the results of Spearman’s correlation and Friedman’s test also inferred that compatibility and clarity of icon elements are the main factors determining a particular icon’s preferability. This study is the first comprehensive study to evaluate the icons associated with the COVID-19 prevention measures. The findings of this study can be utilized as the basis for redesigning icons, particularly for icons related to COVID-19 prevention measures. Furthermore, the approach can also be applied and extended for evaluating other medical icons.

## 1. Introduction

Icons have been widely utilized as a tool to promote COVID-19 prevention measures during the pandemic. They are intended as tools to represent complex information quickly and clearly regarding functions under COVID-19 preventive measures. Upon using them, the goal is to enable visual communication that allows easier transmission of ideas and information compared to written communication [1]. Effective icon designs can overcome language barriers and can successfully convey useful information since they reduce translation requirements and give the information behind them an international look [2].

As for COVID-19 prevention icons, their success and effectivity may have a big impact with regards to virus containment. The best way to prevent illness from the COVID-19 virus is to understand how the virus spreads and to avoid being exposed to it [3]; therefore, accurately understanding and recognizing what functions under COVID-19 preventive measures the visual icons are representing is important. Furthermore, factors that may influence the effectiveness of these icons should be taken into consideration, such as the icon formats [4].

Chi and Dewi [4] classified icons under seven format categories, namely, image-related, concept-related, semi-abstract, word, abbreviation, and combined. Image-related icons are the typical representations of the object or action, concept-related icons represent concepts that are close to but not exactly the concrete image of the action or object, and arbitrary icons have no obvious reference to their intended meaning and can only be meaningful and understood through education [4,5,6,7]. Semi-abstract icons, on the other hand, are combined image-related (concrete representation of an action or object) icons and concept-related or arbitrary icons (an abstract representation of an action or object) [8]. Aside from graphical icons, textual and combined icons are also considered as icon format categories if textual elements are added into the icons [9]. Furthermore, textual icons can be divided into two classifications—word and abbreviation. Because of the obvious mapping of the image-related icon to its referent, it is superior for fast and accurate recognition, while textual icons are better for reaction time [10,11,12,13]. Other graphical icon formats that are concept-related and arbitrary are harder to immediately understand since they have less obvious connection to their intended meaning. Arbitrary icons should be avoided because the need to educate people first to comprehend their meaning requires a considerable amount of funding and time [2].

**Figure 1:**
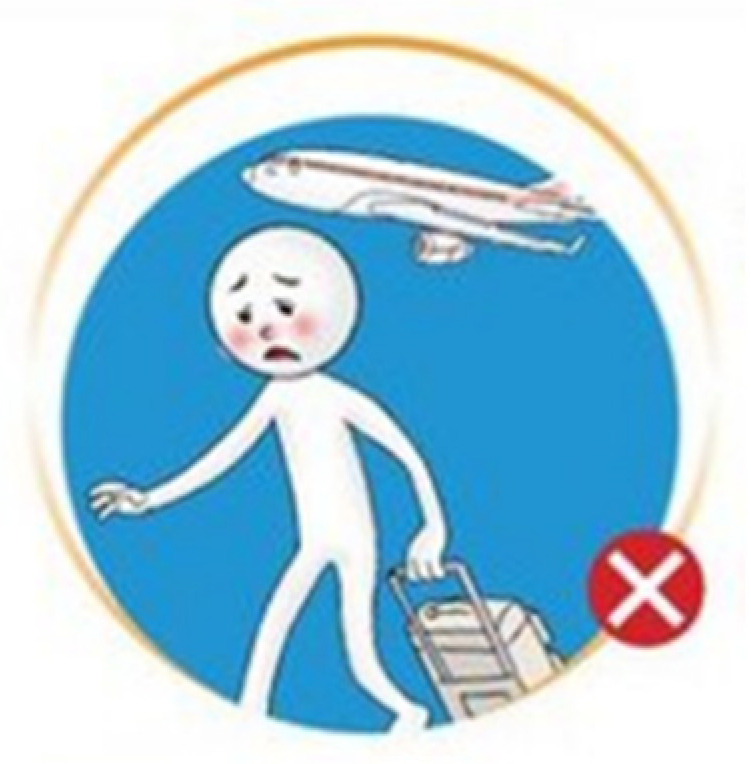
An icon from World Health Organization that intends to convey protocols about travel restriction during the COVID-19 pandemic.

The majority of the existing COVID-19 prevention icons based on the infographics being released by the World Health Organization and other medical or health organizations of different countries are in image- and concept-related formats. For example, the icons that intend to convey protocols about travel restrictions mostly use a concrete illustration of bags or suitcases or a person pulling baggage as a representation (Figure 1). Semi-abstract icon formats were also applied on some preventive measures’ functions. On semi-abstract travel restriction icons, aside from the concrete pictorial representations of the intended meaning, arbitrary symbols are applied [8]. There are icons with a red-colored circle around it or a punctuation mark in it, representing that it is prohibited or that it should be avoided. However, given the advantages and contribution that the visual icons are providing in raising awareness about COVID-19 prevention, there are instances in which the icons used to represent a COVID-19 preventive measure may cause confusion and incorrect comprehension, especially considering icons with a similar context (e.g., the confusion between the icon for shortness of breath and the one for breathing difficulty). The image element of the icon is the major cause of confusion among its readers. In this case, designers should consider and evaluate the icon characteristics to determine the icon’s recognition performance.

Icon characteristics can be classified into physical (external) and psychophysical (internal). Previous studies about icon physical/external characteristics such as size [14], color [15,16], spacing, and density [15] provided a number of practical design guidelines for icon design. Nonetheless, to address the semantic information conveyed by different icons, other studies focused on the influence of icon internal characteristics [17], widely known as psychophysics in human factors and ergonomics. For example, subjective evaluation methods were utilized by Ng and Chan to explore the effects of sign design characteristics on the comprehensibility of traffic and safety signs [18,19]. On the other hand, McDougall et al. carried out a series of studies regarding icon identification to investigate how these characteristics affect users’ cognitive performance [20,21,22,23,24]. Findings on these suggested that there are actually factors that may modify the performance of the icon, such as its communicativeness, complexity, layout, and semantic distance. These were supported by studies that demonstrated that simple and less complex icons can be more easily recognized [2,25,26,27] and concrete icons can be identified more accurately and quickly by the users [12,28]. Complexity pertains to the details intricated on the icon [19], while semantic distance is the measure of the closeness of what is illustrated in the icon to its true intended meaning [19]. Furthermore, it is also suggested that an icon may perform better if it can express its intended message clearly and if its features were arranged carefully [21].

Despite the wide and frequent application of visual icons as a medium for visual communication for COVID-19, no study has yet existed that is mainly about existing COVID-19 prevention icons. In accordance with the International Standards Organization [29], it is necessary to develop icon design principles to ensure visual clarity and subjective preference for enhancing icon recognition and usability. Moreover, there is a lack of study regarding the evaluation of the perceived quality of existing medical-related icons for the broad population and not limited to medical staff.

The purpose of this study is to analyze the existing icons of COVID-19 prevention measures published by the health and medical organizations of different countries. A ranking test and a subjective rating test were utilized to evaluate the collected icons. This study is the first study to analyze the effectiveness of existing icons for COVID-19 prevention measures. The findings are beneficial for human factors engineers, industrial designers, and the even government, particularly for designing medical-related icons.

## 2. Material and Methods

### 2.1. Participants

A total of 57 participants aged between 18 and 40 years old were recruited to participate in this study (Table 1). All of them were the residents of the National Capital Region (Manila), Philippines. Since the data collection was conducted during the COVID-19 pandemic, this study was also conducted in accordance with the Department of Health—Philippines by following COVID-19 safety protocols.

As imposed by the National Ethical Guidelines for Health and Health-Related Research 2017 by the Philippine Health Research Ethics board, all participants were fully informed of the purpose of the research as well as all the procedures within the experiments. The respondents were also asked to complete a consent form before performing the required tasks. Finally, they were also paid 200 PHP after completing the experiment.

### 2.2. Icon Collection

Seven existing icons representing each of the considered COVID-19 preventive measures functions were collected from the COVID-19 prevention infographics released by the Department of Health (DOH) Philippines, World Health Organization (WHO), European Centre for Disease Prevention and Control, and other medical organizations. A total of 133 icons representing the preventive measures of COVID-19 were evaluated and assessed through a ranking test experiment [30]. This was then followed by a subjective rating test for the top two icons of each respondent from the ranking test. The online experiment was posted and distributed through social media platforms. Table 2 shows the nineteen function names of icons related to COVID-19 preventive measures, while all the icons collected for the current study are displayed in Table 3.

### 2.3. Ranking Test

**Figure 2:**
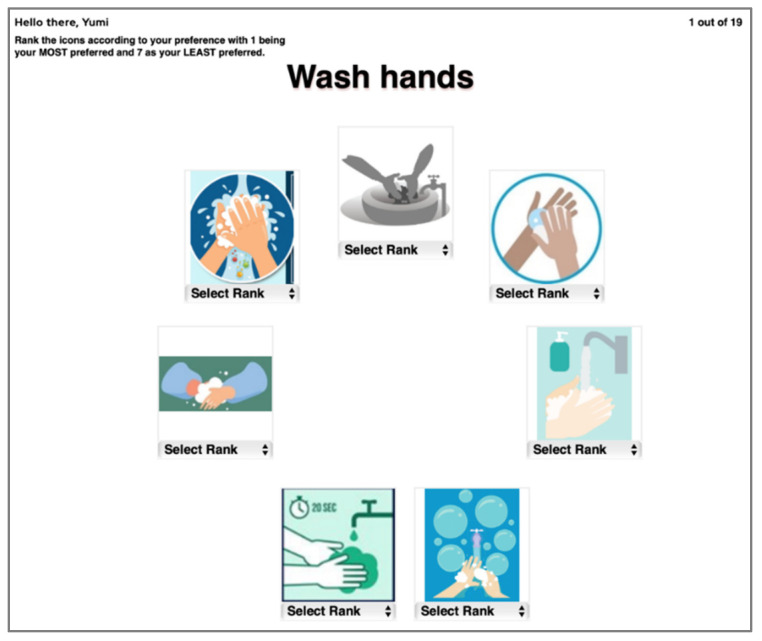
Example of ranking test screen.

In the first phase of the experiment, participants were tasked to rank COVID-19 preventive measure icons within the same function name. Following Chi and Dewi [4], the experiment was administered with a computer program developed using JavaScript and PHP software where respondents ranked the displayed icons from 1 to 7 (See Figure 2). Each participant would rank the most preferred icon under a function name as 1; the next preferred would be ranked as 2, and so on. Thus, the least favored icon was ranked as 7. The icons were laid out in a circular manner to avoid possible sequence effect [31,32] or location bias [33]. The function names were also stated next to the displayed icon to provide appropriate context and description for each function [34]. The experiment was conducted online.

### 2.4. Subjective Rating Test

According to Liu and Ho [35], subjective rating features are reliable in determining the performance of icons based on recognition accuracy [35]. Additionally, subjective scales are easy to administer since they are more sensitive than objective measurements [36]. Therefore, in this phase of the experiment, participants were asked to rate their top 2 icons from the ranking test (i.e., icons with first and second rank for each function) on the basis of subjective design features such as perceived icon quality, communicativeness [21], layout [21], and complexity and semantic distance [4,19,20], as defined in Table 4. Following Chi et al. [37], semantic scales were then assigned for each of the subjective design features (Table 5).

**Figure 3:**
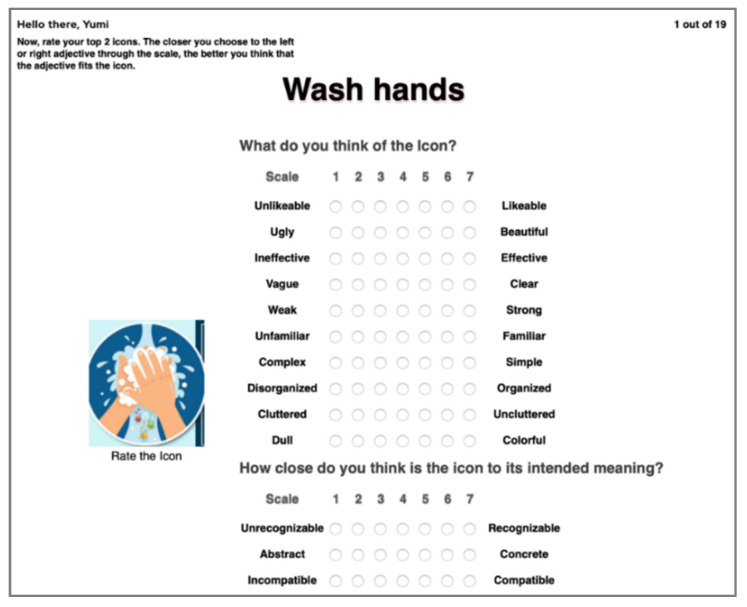
Example of subjective design feature test screen.

The respondents’ top two icons were shown one by one and they were instructed to evaluate the appearance of each icon according to the semantic scales (Figure 3). They were made aware that on the 7-point Likert scale, the closer they choose to the left or right semantic scale, the better they think that the icon displayed fits the semantic scale. However, if they choose the middle of the scale, their opinion of the icon fits both semantic scales. Similar to the ranking test, the test on subjective design features was also developed using JavaScript and PHP software and conducted online.

### 2.5. Statistical Methods

Spearman’s correlation analysis could help readers to find possible correlations between ranking test and the semantic scales. The users’ ranking results were dummy coded 1 for the top ranking and 2 for the second ranking. We hypothesized negative correlations between the ranking test and the semantic scales since more positive semantic scales would lead to better ranking (rank 1 instead of 2). *p* < 0.05 (two-tailed) was set as the threshold for this statistical analysis.

Further detailed analyses for each of 19 functions were conducted using the Friedman’s test. The Friedman’s test was performed to determine the true difference between ranking in terms of each semantic scale to provide a fully meaningful interpretation of the data. It also helps scholars in designing or choosing pertinent communication icons related to COVID-19 prevention measures. As an example, the true difference between ranking 1 and ranking 2 in terms of semantic number 1 (unlikable–likable) for function number 8 (cover when coughing or sneezing) can be evaluated by the following hypotheses:

**Hypothesis** **0** **(H0).** *No true difference between ranking 1 and ranking 2 in terms of “unlikable–likable” for function “cover when coughing or sneezing”.*

**Hypothesis** **1** **(H1).** *There was true difference between ranking 1 and ranking 2 in terms of “unlikable–likable” for function “cover when coughing or sneezing”.*

These two hypotheses were applied for any of the possible conditions (difference between ranking 1 and ranking 2 in terms of each semantic scale to be applied for all the tested functions).

## 3. Results

Table 3 shows the ranking of the icons per function name based on the responses of the participants. The icons were tabulated with their corresponding mean ranking score and its standard deviation The list of icons in Table 3 were sorted based on the mean ranking values. As presented in Table 3, the image-related and combined icon design formats were preferred by the majority of participants, with the image-related format being ranked first on eight of the nineteen function names, and the combined format also obtaining the first ranks on another eight function names. For the remaining three function names, it was the semi-abstract format that was chosen to be first in the rank.

Table 6 shows the descriptive statistics of the semantic scales for all the tested icons. On average, all the tested icons were rated around the score of 6 (of 1–7 scale) for all the subjective design features. These results indicated that all the tested icons are sufficiently recognizable, compatible, organized, simple, familiar, effective, concrete, likeable, clear, uncluttered, strong, beautiful, and colorful.

Table 7 shows the result of the Spearman’s correlation analysis (two-tailed) between pairs of the semantic scales and the ranks for all functions. The analysis showed that the 13 semantic scales were significantly intercorrelated to each other. Although all semantic scales were also significantly correlated with the ranks, we can highlight that vague–clear, weak–strong, and incompatible–compatible were the three ones with the highest correlation coefficients, i.e., −0.206, −0.205, and −0.200, respectively.

Table 8 represents the Friedman’s test result for determining the true difference between ranking 1 and ranking 2 in terms of “unlikable–likable” for function “cover when coughing or sneezing”. Based on this table, the mean rank for unlikable–likable rank 1 was 1.7 while the mean rank for unlikable–likable rank 2 was 1.3. Chi-square statistic indicated that there was a significant difference between ranking 1 and ranking 2 in terms of “unlikable–likable” (Table 8). In other words, H0 was nullified and H1 was true.

Table 9 represents the summary of all the Friedman’s tests on each semantic scale for all functions. Based on this table, we can see that there were some significant differences between ranking 1 and ranking 2 in different semantic scales. Similar results were gathered for Spearman’s and Friedman’s tests (See Table 9), particularly for weak–strong and incompatible–compatible, where these two semantic scales showed a significant difference between ranking 1 and ranking 2 in 11 out of 19 functions. On the other side, Friedman’s tests showed that for function 1 (Shortness of Breath), Function 5 (Wash Hands), and Function 16 (Wash Clothes Properly), all the semantic scales tested in this study did not have a significant difference between ranking 1 and ranking 2.

## 4. Discussion

From the ranking test results, it can be concluded that icon users prefer the icons to be in image-related formats. However, for some function names (e.g., shortness of breath and difficulty in breathing), even though the icons are in image-related format, they were still ranked low if the images in the icon were drawn in silhouette-like illustrations, indicating that the users probably like image-related icons only if they are illustrated realistically or in a more concrete way. This may be because the concreteness of the icons improves its ability to convey its meaning, as concrete symbols tend to be more visually obvious since they depict objects, places, and people that are already familiar to us in the real world [41,42]. Therefore, the more concrete the icon is, the better the semantic distance is, and this results in its users being able to react quickly and accurately to it [12,28]. The combined format, which is a combination of icons and textual labels [10], is also at the same level as the image-related format in terms of the number of function names for which it was ranked first. While image-related icons are known to be superior for fast and accurate recognition because of the obvious mapping between the icon and its referent [10,11,12], textual icons, on the other hand, are proven to be better for reaction time [13]. Since the combined format is the combination of the icons and textual labels [10], the reaction and recognition accuracy of the users to these icons could be significantly increased. In the current study, it is noticeable that if image-related icons are incorporated with textual labels, making it a combined format, the icon users favor them. Furthermore, despite the guideline given by the International Standards Organization [29] that the use of abstract symbols should be avoided, results of this study demonstrated that semi-abstract icons, which are combined image-related (concrete representation of an action or object) icons and concept-related or arbitrary icons (abstract representation of an action or object) [8], are still the most preferred on three function names—a result that is similar to that of Chi et al. [5]. On the functions “avoid touching face” and “avoid travelling to places with known cases”, the red circle around the icons and the cross marks might have helped in relating them to their correct function names.

Concept-related and arbitrary icon design formats had consistently low ranking scores. This is understandable considering that concept-related icons outline concepts that are close but are still not the exact concrete image of the action or object, and arbitrary icons have no clear reference to their intended meaning and can only be meaningful and understood through education [4,5,6,7]. As a result, compared to the formats that obtained good ranking scores, the icons designed in these two formats do not have obvious mappings to their referent. Therefore, the connection of the icons designed in these formats to their corresponding function names is harder to distinguish, making them the least favored formats for the users.

The result of Spearman’s correlation and Friedman’s tests infers that compatibility and clarity of icon elements are the main factors determining a particular icon’s preferability. Moreover, the alternative icons with stronger communicativeness in delivering the message should be prioritized to be implemented. Furthermore, the high and significant intercorrelations of Spearman’s test (See Table 7) between weak–strong, ineffective–effective, and vague–clear also suggest the importance of selecting icons with better clarity to effectively and powerfully deliver the messages related to COVID-19 prevention measures. However, this study also reveals that for specific functions, there is no significant relationship between preference and tested semantic scales, inferring the possibility of inclusion of other semantic scales in the future study.

Despite the clear contributions of the study for design guidelines on COVID-19 prevention measures icons, the researchers would like to acknowledge several limitations of this study. First, because the study was facilitated during the middle of a pandemic, the authors resorted to conducting the data gathering online, and the online experiment was answered by a total of 57 participants. To produce more comprehensive results, future researchers may consider increasing the number of respondents. It is also recommended to broaden the scope of the current study to come up with more thorough and specific design references for COVID-19 preventive measures icons. Second, this study only focused on knowing and understanding the preference of the icon users through the ranking test and preference test that were performed. Aside from considering how the icons satisfy the subjective preferences of the users, future research should also incorporate the performance of the icons in terms of the effectiveness to accurately interpret their corresponding function names. Finally, future study should utilize eye-tracker [43] to find the relationship between the results of ranking test and eye movement behavior to provide more meaningful findings.

## 5. Conclusions

Icon has been widely utilized as a tool to promote COVID-19 prevention measures. The purpose of this study was to analyze 133 existing icons of COVID-19 prevention measures published by the health and medical organizations of different countries.

A rank ordering test was conducted for the seven icons representing each function name, followed by a subjective rating test for the top two chosen icons of the respondent form the ranking test. Generally, findings from the current study showed that the image presented in the icon is the key point on which the perceived quality of the icon depends [44,45], and the preference of users for the icon may rely on this. In this case, designers may consider the cognitive features of an icon such as its familiarity, its concreteness, the complexity of the design intricated on it, its meaningfulness, and its semantic distance or its closeness to its intended meaning [20,46,47].

The current study also further proves that that familiarity and semantic distance should be of primary importance when it comes to selecting icons [22,39]. Interestingly, Spearman’s correlation analysis between ranking and semantic scales showed that incompatible–compatible, vague–clear, weak–strong, and abstract–concrete were the four strongest semantic scales that highly correlated with the preference ranking. In addition, Friedman’s tests inferred that compatibility and clarity of icon elements are the main factors determining a particular icon’s preferability. These suggest that designers should choose images that are realistic and as closely related as possible to the function represented by the icon. It should also be simple and straightforward to reduce complexity. Adding elements on graphical or image-related icons, whether textual or arbitrary symbols, is recommendable since they may increase the cognition of the users into the icons and therefore can make them preferable. Icon design formats having less connection to what they actually depict, such as the concept-related and arbitrary formats, should be avoided since they are more challenging to comprehend and are probably not preferred. Another observable result of the study is that color also affects how well liked the icons are. Black and white and grayscale icons obtained low ranking scores, even though they concretely represent their function name. This gives the conclusion that designers should also consider making the icons colorful so that they may be more visually appealing and likeable.

This study is the first comprehensive study to evaluate the icons associated with the COVID-19 prevention measures. The findings of this study can be utilized as the basis for redesigning icons, particularly for icons related to COVID-19 prevention measures [48]. Furthermore, the approach can also be applied and extended for evaluating other medical icons [49,50], safety icons, disaster-related prevention icons [51], transportation-related icons [52,53], and even entertainment-related icons [54,55,56,57,58].

## Figures and Tables

**Table 1:** The demographic of the participants (N = 57).

Characteristics	Category	N	%
Gender	Male	25	43.9
	Female	32	56.1
Age	18–25	48	84.2
	26–33	3	5.3
	34–40	6	10.5
Educational Background	High School Graduate	18	31.6
	College Graduate	36	63.2
	Master Graduate	3	5.3

**Table 2:** Function names of icons related to COVID-19 preventive measures.

Functions
1. Shortness of Breath
2. Fever
3. Cough or Cold
4. Difficulty in Breathing
5. Wash Hands
6. Consult Doctor/Seek Medical Help
7. Avoid People with Flu-Like Symptoms
8. Cover Face when Coughing/Sneezing
9. Get Information from Trusted Sources
10. Wear Face Mask
11. Avoid Crowded Places/Limit Social Gatherings
12. Dispose Tissue and Face Mask in Waste Can
13. Use Alcohol-Based Hand Sanitizers if Soap and Water are Not Available.
14. Avoid Travelling to Places with Known Cases
15. Avoid Touching your Face
16. Wash Clothes Properly
17. De-Contaminate/Disinfect
18. Home Quarantine/Stay at Home
19. Social Distancing

**Table 3:** Icons tested in the current study and their summarized results from the preference test.

Function	Rank 1	Rank 2	Rank 3	Rank 4	Rank 5	Rank 6	Rank 7
Shortness of Breath (1)	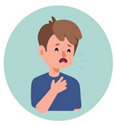 (mean: 2.807; SD 1.652)	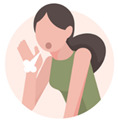 (mean: 3.175; SD: 1.843)	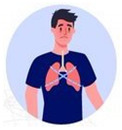 (mean: 3.351; SD: 1.747)	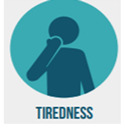 (mean: 4.351; SD: 1.986)	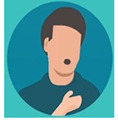 (mean: 4.386; SD: 2.042)	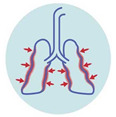 (mean: 4.93; SD: 1.981)	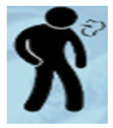 (mean: 5.000; SD: 1.592)
Source	(k)	(j)	(c)	(e)	(e)	(l)	(a)
Fever (2)	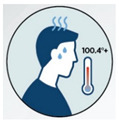 (mean: 2.772; SD: 1.604)	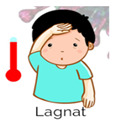 (mean: 2.807; SD: 1.903)	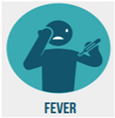 (mean: 3.509; SD: 1.774)	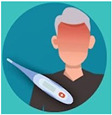 (mean: 3.719; SD: 1.556)	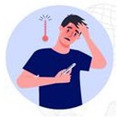 (mean: 3.737; SD: 1.395)	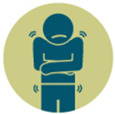 (mean: 5.509; SD: 1.692)	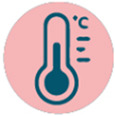 (mean: 5.947; SD:1.54)
Source	(m)	(a)	(e)	(e)	(c)	(e)	(e)
Cough or Cold (3)	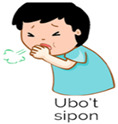 (mean: 2.789; SD: 1.75)	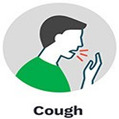 (mean: 2.86; SD: 1.575)	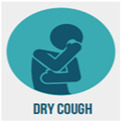 (mean: 3.158; SD: 1.601)	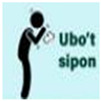 (mean: 3.298; SD: 1.669)	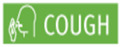 (mean: 4.93; SD: 1.438)	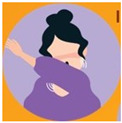 (mean: 5.263; SD: 1.685)	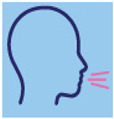 (mean: 5.702; SD: 1.792)
Source	(a)	(n)	(e)	(a)	(a)	(d)	(a)
Difficulty in Breathing (4)	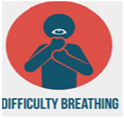 (mean: 2.772; SD: 1.991)	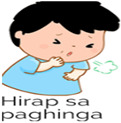 (mean: 3.333; SD: 1.786)	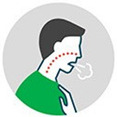 (mean: 3.86; SD: 1.652)	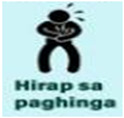 (mean: 3.86; SD: 1.726)	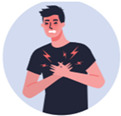 (mean: 4.07; SD: 2.017)	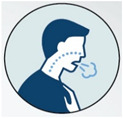 (mean: 4.105; SD: 1.961)	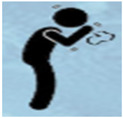 (mean: 6.000; SD: 1.268)
Source	(e)	(a)	(n)	(a)	(b)	(m)	(a)
Wash Hands (5)	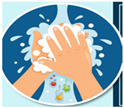 (mean: 2.526; SD: 1.91)	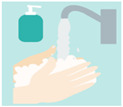 (mean: 2.895; SD: 1.622)	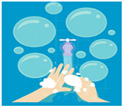 (mean: 3.544; SD: 1.477)	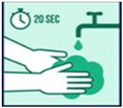 (mean: 3.544; SD: 1.753)	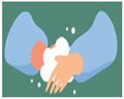 (mean: 4.579; SD: 1.569)	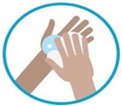 (mean: 4.614; SD: 1.556)	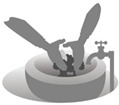 (mean: 6.298; SD: 1.451)
Source	(o)	(d)	(e)	(b)	(d)	(d)	(h)
Consult Doctor/Seek Medical Help (6)	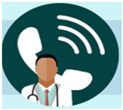 (mean: 1.789; SD: 1.176)	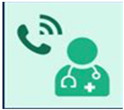 (mean: 2.965; SD: 1.488)	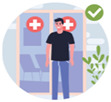 (mean: 3.439; SD: 1.604)	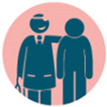 (mean: 3.807; SD: 1.563)	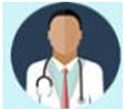 (mean: 3.965; SD: 1.5)	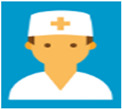 (mean: 5.386; SD: 0.94)	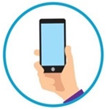 (mean: 6.649; SD: 1.11)
Source	(d)	(b)	(d)	(e)	(a)	(d)	(d)
Avoid People with Flu-Like Symptoms (7)	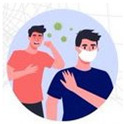 (mean: 2.439; SD: 1.239)	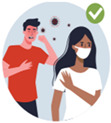 (mean: 2.491; SD: 1.571)	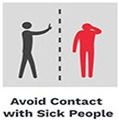 (mean: 2.702; SD: 1.792)	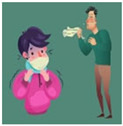 (mean: 4.228; SD: 1.268)	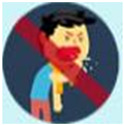 (mean: 4.807; SD: 1.608)	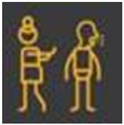 (mean: 5.298; SD: 1.488)	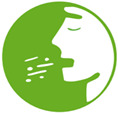 (mean: 6.035; SD: 1.309)
Source	(d)	(d)	(d)	(a)	(a)	(d)	(g)
Cover when Coughing or Sneezing (8)	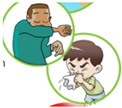 (mean: 2.667; SD: 2.003)	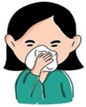 (mean: 3.000; SD: 1.5)	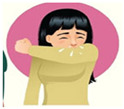 (mean: 3.404; SD: 1.811)	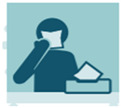 (mean: 4.246; SD: 1.735)	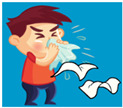 (mean: 4.316; SD: 1.649)	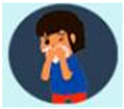 (mean: 4.614; SD: 1.634)	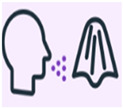 (mean: 5.754; SD: 1.921)
Source	(a)	(d)	(d)	(e)	(a)	(a)	(d)
Get Information from Trusted Sources (9)	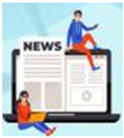 (mean: 1.614; SD: 1.013)	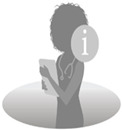 (mean: 3.158; SD: 1.544)	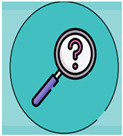 (mean: 3.316; SD: 1.844)	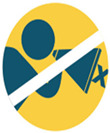 (mean: 4.316; SD: 1.919)	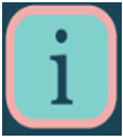 (mean: 4.895; SD: 1.41)	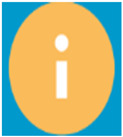 (mean: 5.018; SD: 1.458)	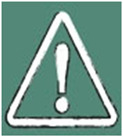 (mean: 5.684; SD: 1.429)
Source	(d)	(h)	(e)	(e)	(a)	(a)	(a)
Wear Face Mask (10)	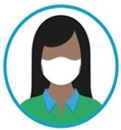 (mean: 2.877; SD: 1.722)	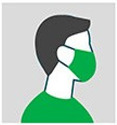 (mean: 2.982; SD: 1.685)	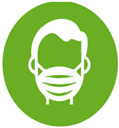 (mean: 3.158; SD: 1.177)	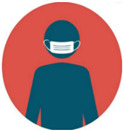 (mean: 3.579; SD: 1.625)	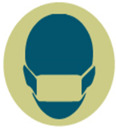 (mean: 3.579; SD: 1.658)	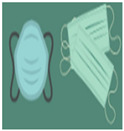 (mean: 5.702; SD: 2.009)	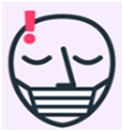 (mean: 6.123; SD: 0.965)
Source	(p)	(n)	(g)	(e)	(e)	(a)	(d)
Avoid Crowded Places/Limit Social Gatherings (11)	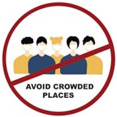 (mean: 1.754; SD: 1.023)	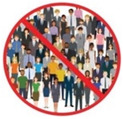 (mean: 2.211; SD: 1.333)	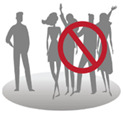 (mean: 3.754; SD: 1.418)	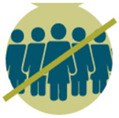 (mean: 3.947; SD: 1.231)	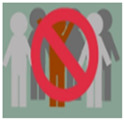 (mean: 4.193; SD: 1.381)	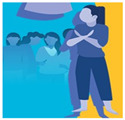 (mean: 5.439; SD: 1.35)	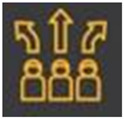 (mean: 6.702; SD: 0.626)
Source	(d)	(d)	(h)	(e)	(d)	(d)	(d)
Dispose Tissue and Face Mask in Waste Can (12)	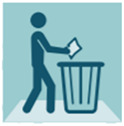 (mean: 1.86; SD: 1.025)	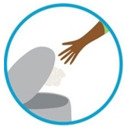 (mean: 2.404; SD: 1.237)	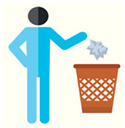 (mean: 2.456; SD: 1.351)	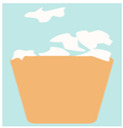 (mean: 4.579; SD: 1.535)	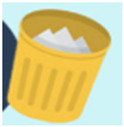 (mean: 5.158; SD: 0.996)	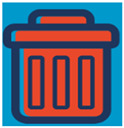 (mean: 5.439; SD: 1.165)	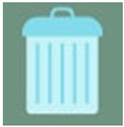 (mean: 6.105; SD: 1.175)
Source	(e)	(q)	(d)	(f)	(d)	(d)	(a)
Use Hand Sanitizers (13)	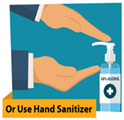 (mean: 1.386; SD: 0.861)	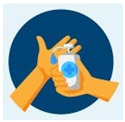 (mean: 3.228; SD: 1.402)	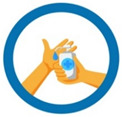 (mean: 3.649; SD: 1.395)	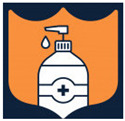 (mean: 4.351; SD: 1.631)	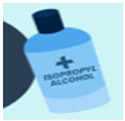 (mean: 4.772; SD: 1.722	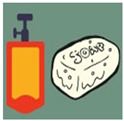 (mean: 5.298; SD: 1.812)	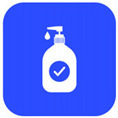 (mean: 5.316; SD: 1.744)
Source	(e)	(r)	(d)	(i)	(d)	(a)	(i)
Avoid Traveling to Places with Known Cases (14)	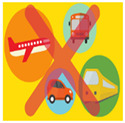 (mean: 1.807; SD: 1.302)	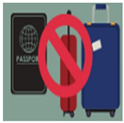 (mean: 2.702; SD: 1.721)	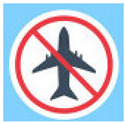 (mean: 3.702; SD: 1.463)	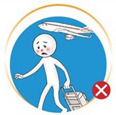 (mean: 3.86; SD: 1.505	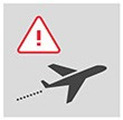 (mean: 4.14; SD: 1.302)	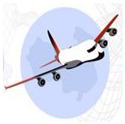 (mean: 5.877; SD: 1.001)	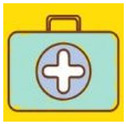 (mean: 5.912; SD: 1.64)
Source	(d)	(a)	(s)	(d)	(d)	(d)	(d)
Avoid Touching Face (15)	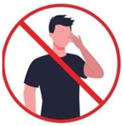 (mean: 2.193; SD: 1.517)	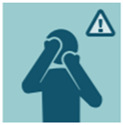 (mean: 2.93; SD: 1.689)	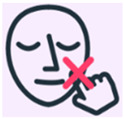 (mean: 3.281; SD: 1.77	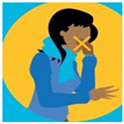 (mean: 3.404; SD: 1.438)	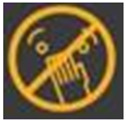 (mean: 5.105; SD: 1.372)	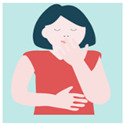 (mean: 5.14; SD: 1.381)	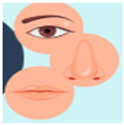 (mean: 5.947; SD: 1.597)
Source	(d)	(e)	(d)	(d)	(d)	(f)	(d)
Wash Clothes Properly (16)	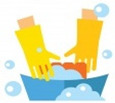 (mean: 2.667; SD: 1.704)	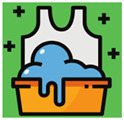 (mean: 2.579; SD: 1.569)	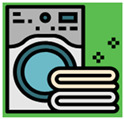 (mean: 2.825; SD: 1.627)	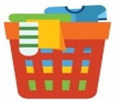 (mean: 3.842; SD: 1.556	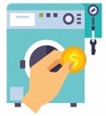 (mean: 4.684; SD: 1.416)	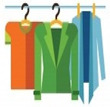 (mean: 4.912; SD: 1.455	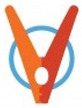 (mean: 6.491; SD: 1.002)
Source	(i)	(d)	(d)	(i)	(i)	(i)	(i)
De-Contaminate (17)	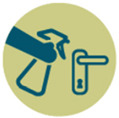 (mean: 2.526; SD: 1.477)	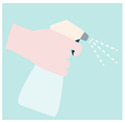 (mean: 3.263; SD: 1.904)	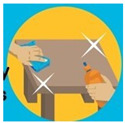 (mean: 3.86; SD: 1.807)	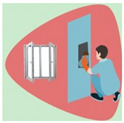 (mean: 4.035; SD: 2.026)	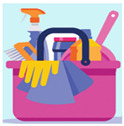 (mean: 4.07; SD: 2.17)	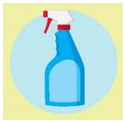 (mean: 4.947; SD: 1.807)	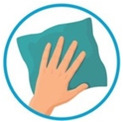 (mean: 5.298; SD: 1.388
Source	(e)	(d)	(d)	(d)	(d)	(d)	(q)
Home Quarantine/Stay at Home (18)	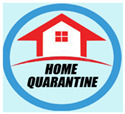 (mean: 1.439; SD: 0.982)	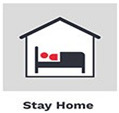 (mean: 2.614; SD: 1.221)	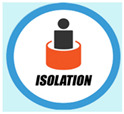 (mean: 3.368; SD: 1.588)	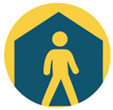 (mean: 4.596; SD: 1.545)	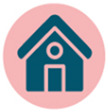 (mean: 5.035; SD: 1.752)	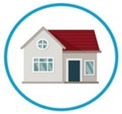 (mean: 5.263; SD: 1.232)	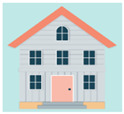 (mean: 5.684; SD: 1.242)
Source	(d)	(d)	(d)	(e)	(e)	(q)	(f)
Social Distancing (19)	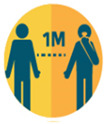 (mean: 2.158; SD: 1.192)	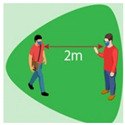 (mean: 2.281; SD: 1.485	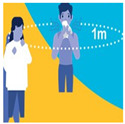 (mean: 3.526 SD: 1.67)	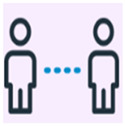 (mean: 4.684; SD: 1.774)	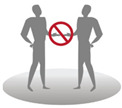 (mean: 4.737; SD: 1.653)	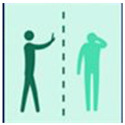 (mean: 5.263; SD: 1.446)	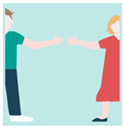 (mean: 5.351; SD: 1.706)
Source	(e)	(d)	(d)	(d)	(h)	(b)	(a)

Note: (a) Department of Health, Philippines, (b) Department of National Defense, Philippines, (c) Makati Medical Center, Philippines, (d) World Health Organization, (e) Pan American Health Organization, United State of America, (f) Health Direct, Australia, (g) European Centre for Disease Prevention and Control, Europe, (h) World Heart Federation, (i) Freepik.com, (j) Driblle.com, (k) Margaret Mary Health, (l) San Joaquin Country Public Health Services, (m) Sanford Health Organization, (n) Lompoc Valley Medical Center, (o) Wyckoff Family YMCA, (p) MIT College of Pharmacy Moradabad, (q) Iskandar Malaysia Studios, (r) Sai Sanjeevini Hospitals, and (s) Center for Disease Control and Prevention.

**Table 4:** Descriptions of subjective design features.

Subjective Design Features	Definition
Perceived Icon Quality	One of the most critical aspects of icon development that defines the successful design [38]
Communicativeness	Refers to how the icon expresses its intended meaning [21].
Complexity	Pertains to how complex the details intricated on the icon are [19].
Layout	How carefully the features of an icon are arranged [21].
Semantic Distance	The measure of the closeness of what is illustrated in the icon to its true intended meaning [20].

**Table 5:** Semantic scales and their corresponding subjective design feature.

Subjective Design Features	Semantic Scales
Perceived Icon Quality	1. Unlikable—Likable 2. Ugly—Beautiful [39] 3. Ineffective—Effective
Communicativeness	4. Vague—Clear 5. Weak—Strong [39] 6. Unfamiliar—Familiar [4,21,39]
Complexity	7. Complex—Simple [4,7,40]
Layout	8. Disorganized—Organized 9. Cluttered—Uncluttered 10. Dull—Colorful [39]
Semantic Distance	11. Unrecognizable—Recognizable [21] 12. Abstract—Concrete [4,39] 13. Incompatible—Compatible

**Table 6:** Descriptive statistics: semantic scales.

Variable	Mean	SD
1. unlikeable–likeable	6.0651	1.2062
2. ugly–beautiful	5.8347	1.2821
3. ineffective–effective	6.0803	1.2331
4. vague–clear	6.0416	1.278
5. weak–strong	5.9861	1.2754
6. unfamiliar–familiar	6.0974	1.2317
7. complex–simple	6.1053	1.2513
8. disorganized–organized	6.1283	1.1324
9. cluttered–uncluttered	5.9908	1.27
10. dull–colorful	5.7664	1.4093
11. unrecognizable–recognizable	6.1768	1.1496
12. abstract–concrete	6.0794	1.1405
13. incompatible–compatible	6.1491	1.1313

**Table 7:** Spearman’s rank correlation result between ranking and 13 semantic scales.

		1	2	3	4	5	6	7	8	9	10	11	12	13
Unlikable–Likable	1	1												
Ugly–Beautiful	2	0.749 **	1											
Ineffective–Effective	3	0.645 **	0.610 **	1										
Vague–Clear	4	0.584 **	0.567 **	0.786 **	1									
Weak–Strong	5	0.609 **	0.607 **	0.787 **	0.796 **	1								
Unfamiliar–Familiar	6	0.560 **	0.553 **	0.709 **	0.722 **	0.749 **	1							
Complex–Simple	7	0.494 **	0.489 **	0.661 **	0.666 **	0.689 **	0.727 **	1						
Disorganized–Organized	8	0.579 **	0.569 **	0.684 **	0.681 **	0.685 **	0.709 **	0.774 **	1					
Cluttered–Uncluttered	9	0.548 **	0.560 **	0.637 **	0.621 **	0.635 **	0.655 **	0.731 **	0.819 **	1				
Dull–Colorful	10	0.552 **	0.619 **	0.454 **	0.448 **	0.471 **	0.442 **	0.362 **	0.460 **	0.448 **	1			
Unrecognizable–Recognizable	11	0.586 **	0.526 **	0.706 **	0.701 **	0.682 **	0.661 **	0.627 **	0.639 **	0.604 **	0.428 **	1		
Abstract–Concrete	12	0.575 **	0.547 **	0.703 **	0.700 **	0.706 **	0.653 **	0.634 **	0.656 **	0.632 **	0.459 **	0.752 **	1	
Incompatible–Compatible	13	0.587 **	0.542 **	0.707 **	0.681 **	0.693 **	0.653 **	0.613 **	0.650 **	0.622 **	0.453 **	0.784 **	0.816 **	1
Rank		−0.195 **	−0.134 **	−0.199 **	−0.206 **	−0.205 **	−0.169 **	−0.162 **	−0.176 **	−0.152 **	−0.145 **	−0.194 **	−0.195 **	−0.200 **

**. Correlation is significant at the 0.01 level (two-tailed).

**Table 8:** The Friedman’s test result in terms of “unlikable–likable” for function “cover when coughing or sneezing”.

Ranks	
	Mean Rank
Unlikable–Likable Rank 1	1.7
Unlikable–Likable Rank 2	1.3
**Test Statistics**	
N	57
Chi-Square	14.297
df	1
Asymptotic significance	0.000

**Table 9:** The summary results of the Friedman’s test in chi-square statistic (χ^2^).

	1	2	3	4	5	6	7	8	9	10	11	12	13
Function 1	0.024	0.9	0.947	0.1	0.021	1.884	0	2.273	0.364	1.089	3.27	0.1	1.256
Function 2	7.049 **	6.149 **	1.882	6.721 **	6.422 **	2.5	4 *	4.667 *	3.13	4.787 **	6.081 **	7.811 **	6.4 **
Function 3	5.818 **	1.256	5.488 **	13.714 **	8.100 **	4.667 *	0.676	1.778	3.457	3.13	11.765 **	12.6 **	9.323 **
Function 4	4.667 **	1.6	4.568 **	7.714 **	5.818 **	1.195	0	0.857	0.1	2.273	3.789	1.524	4.122 *
Function 5	2	1.4	1.195	2.814	2.951	0.818	0.22	2.189	1.256	0.037	2.5	1.524	0.857
Function 6	1.976	1.976	4.122 *	1.524	0.61	0.231	2.077	2.632	1.195	0	4.333*	1.524	5.233 **
Function 7	2.077	1.976	2.314	0.9	2.778	1.778	0.947	1.195	0.22	6.721 **	0.421	2.077	2.5
Function 8	14.297 **	5.444 **	7.111 **	10.314 **	9.757 **	7.258 **	6.818 **	3.457	6.429 **	11.308 **	13.333 **	14.235 **	15.125 **
Function 9	4.333 **	1.195	5.488 **	2.381	6.721 **	5.233 **	7.049 **	2.189	3.27	1.391	1.684	3.596	2.381
Function 10	6.081 **	1.524	3.333	7.529 **	6.125 **	2.133	6.737 **	9 **	7.346 **	5.444 **	5.121 **	4.235 **	10.125 **
Function 11	0.15	1.4	2	1.778	5.765 **	4.235 **	2	5.143 **	1.2	2.814	2.455	6.533	3.457
Function 12	2.814	0.243	8.805 **	4.333 **	5.333 **	0.857	1.524	2.814	0.206	4.122 **	2.189	3.756	5.769 **
Function 13	6.429 **	1.256	0.4	2.189	3.27	1.485	5.121 **	1.778	3.6	6.081 **	3.103	4.235 **	1.485
Function 14	3.125	0.105	10.526 **	10.314 **	7.111 **	6.737 **	5.121 **	9.757 **	7.111 **	0.231	11.111 **	9 **	4.568 **
Function 15	1.485	1.4	4 **	10.756 **	6.081 **	4.333 **	12.6 **	9 **	4 **	2.778	7.41 **	3.457	6.081 **
Function 16	0.118	0.4	0.111	0.027	0.118	0.758	1.125	0.105	0.22	0.9	3.457	1.324	3.27
Function 17	10.800 **	7.410 **	2.778	1.778	1.6	1.125	1.385	2.286	7.529 **	6.422 **	2.286	5.452 **	5.765 *
Function 18	4.235 *	2.635	8.395 **	7.078 **	5.488 *	4.900*	6.095 *	8.395 **	5.488 *	1.976	1.884	3.130	5.000 *
Function 19	4.333 *	2.951	7.111 **	3.333	2.778	6.818 **	5.765 *	2.793	4.235 *	0.243	1.581	4.568 *	1.690

**. Chi-square statistic is significant at the 0.01 level. *. Chi-square statistic is significant at the 0.05 level (two-tailed). 1: unlikeable–likeable; 2: ugly–beautiful; 3: ineffective–effective; 4: vague–clear; 5: weak–strong; 6: unfamiliar–familiar; 7: complex–simple; 8: disorganized–organized; 9: cluttered–uncluttered; 10: dull–colorful; 11:unrecognizable–recognizable; 12: abstract–concrete; 13: incompatible–compatible.

## Data Availability

The data presented in this study are available on request from the corresponding author.

## References

[1] Vigoroso L., Caffaro F., Cavallo E. (2020). Occupational safety and visual communication: User-centred design of safety training material for migrant farmworkers in Italy. Saf. Sci..

[2] Horton W. (1994). The Icon Book: Visual Symbols for Computer Systems and Documentation.

[3] Centers for Disease Control and Prevention (2020). How to Protect Yourself & Others. https://www.cdc.gov/coronavirus/2019-ncov/prevent-getting-sick/prevention.html.

[4] Chi C.-F., Dewi R.S. (2014). Matching performance of vehicle icons in graphical and textual formats. Appl. Ergon..

[5] Chi C.-F., Dewi R.S., Samali P., Hsieh D.-Y. (2019). Preference ranking test for different icon design formats for smart living room and bathroom functions. Appl. Ergon..

[6] Lodding K. (1983). Iconic Interfacing. IEEE Comput. Graph. Appl..

[7] Goonetilleke R., Shih H.M., On H.K., Fritsch J. (2001). Effects of training and representational characteristics in icon design. Int. J. Hum. Comput. Stud..

[8] Blattner M., Sumikawa D., Greenberg R. (1989). Earcons and Icons: Their Structure and Common Design Principles. Hum. Comput. Interact..

[9] Reder P.J., Mccallum C.A. (2004). Flexible Mouse-Driven Method of User Interface. U.S. Patent.

[10] Wiedenbeck S. (1999). The use of icons and labels in an end user application program: An empirical study of learning and retention. Behav. Inf. Technol..

[11] Blankenberger S., Hahn K. (1991). Effects of icon design on human-computer interaction. Int. J. Man-Mach. Stud..

[12] Stotts D.B. (1998). The Usefulness of Icons on the Computer Interface: Effect of Graphical Abstraction and Functional Representation on Experienced and Novice Users. Proc. Hum. Factors Ergon. Soc. Annu. Meet..

[13] Dewar R.E., Ells J.G., Mundy G. (1976). Reaction Time as an Index of Traffic Sign Perception. Hum. Factors J. Hum. Factors Ergon. Soc..

[14] Lindberg T., Näsänen R. (2003). The effect of icon spacing and size on the speed of icon processing in the human visual system. Displays.

[15] Ojanpää H., Näsänen R. (2003). Effects of luminance and colour contrast on the search of information on display devices. Displays.

[16] Huang K.-C., Chang W.-T., Wei W.-L. (2010). Effects of visual field, exposure time, and set size on icon search with varied delays using an LCD monitor. J. Soc. Inf. Disp..

[17] Shen Z., Zhang L., Li R., Liang R. (2020). The effects of icon internal characteristics on complex cognition. Int. J. Ind. Ergon..

[18] Ng A.W., Chan A.H. (2007). The guessability of traffic signs: Effects of prospective-user factors and sign design features. Accid. Anal. Prev..

[19] Ng A., Chan A. Visual and Cognitive Features on Icon Effectiveness. Proceedings of the International Multiconference of Engineers and Computer Scientists.

[20] McDougall S.J.P., Curry M.B., De Bruijn O. (1999). Measuring symbol and icon characteristics: Norms for concreteness, complexity, meaningfulness, familiarity, and semantic distance for 239 symbols. Behav. Res. Methods Instrum. Comput..

[21] Huang S.-M., Shieh K.-K., Chi C.-F. (2002). Factors affecting the design of computer icons. Int. J. Ind. Ergon..

[22] Isherwood S.J., McDougall S., Curry M.B. (2007). Icon Identification in Context: The Changing Role of Icon Characteristics With User Experience. Hum. Factors J. Hum. Factors Ergon. Soc..

[23] Silvennoinen J.M., Kujala T., Jokinen J.P. (2017). Semantic distance as a critical factor in icon design for in-car infotainment systems. Appl. Ergon..

[24] Tsai C.-Y. (2017). Effect of graphic simplification and graphic metaphor on the memory and identification of travel map. Int. J. Ind. Ergon..

[25] Gittins D. (1986). Icon-based human-computer interaction. Int. J. Man-Mach. Stud..

[26] García M., Badre A.N., Stasko J.T. (1994). Development and validation of icons varying in their abstractness. Interact. Comput..

[27] McDougall S.J.P., de Bruijn O., Curry M.B. (2000). Exploring the effects of icon characteristics on user performance: The role of icon concreteness, complexity, and distinctiveness. J. Exp. Psychol. Appl..

[28] Stammers R., Hoffman J. (1991). Transfer between Icon Sets and Ratings of Icon Concreteness and Appropriateness. Proc. Hum. Factors Soc. Annu. Meet..

[29] International Standards Organization (2007). Graphical Symbols—Creation and Design of Public Information Symbols—Requirements.

[30] Gingold M., Shteingart S., Green P. (1981). Truck Drivers’ Suggestions and Preferences for Instrument Panel Symbols.

[31] Chi C.-F., Drury C. (1988). A further note on psychophysical testing of handles. Appl. Ergon..

[32] Chi C.-F., Dewi R.S., Huang M.-H. (2016). Psychophysical evaluation of auditory signals in passenger vehicles. Appl. Ergon..

[33] Hoekstra E., Williams M., Green P. (1993). Development and Driver Understanding of Hazard Warning and Location Symbols for IVSAWS.

[34] Wolff J.S., Wogalter M.S. (1998). Comprehension of Pictorial Symbols: Effects of Context and Test Method. Hum. Factors J. Hum. Factors Ergon. Soc..

[35] Liu Y.-C., Ho C.-H. (2012). The effects of age on symbol comprehension in central rail hubs in Taiwan. Appl. Ergon..

[36] Chi C.-F., Lin F.-T. (1998). A Comparison of Seven Visual Fatigue Assessment Techniques In Three Data-Acquisition VDT Tasks. Hum. Factors J. Hum. Factors Ergon. Soc..

[37] Chi C.-F., Dewi R.S., Surbakti Y.Y., Hsieh D.-Y. (2017). The perceived quality of in-vehicle auditory signals: A structural equation modelling approach. Ergonomics.

[38] Stylidis K., Wickman C., Söderberg R. (2020). Perceived quality of products: A framework and attributes ranking method. J. Eng. Des..

[39] Bovea M.D., Quemades-Beltrán P., Pérez-Belis V., Juan P., Braulio-Gonzalo M., Ibáñez-Forés V. (2018). Options for labelling circular products: Icon design and consumer preferences. J. Clean. Prod..

[40] Choi J.H., Lee H.-J. (2012). Facets of simplicity for the smartphone interface: A structural model. Int. J. Hum. Comput. Stud..

[41] Campbell J.L., Hoffmeister D.H., Kiefer R.J., Selke D.J., Green P., Richman J.B. (2004). Comprehension Testing of Active Safety Symbols. J. Passeng. Cars Mech. Syst. J..

[42] Rogers Y. (1989). Icons at the interface: Their usefulness. Interact. Comput..

[43] Liu W., Cao Y., Proctor R.W. (2021). How do app icon color and border shape influence visual search efficiency and user experi-ence? Evidence from an eye-tracking study. Int. J. Ind. Ergon..

[44] Cahigas M.M., Prasetyo Y.T. Matching-Based Comprehension of Emergency Safety Symbols Among Filipinos: Us-er-Centered Quality Measure. Proceedings of the 21st Congress of the International Ergonomics Association (IEA 2021).

[45] Madel L., Cahigas M., Tri Prasetyo Y. Kansei Engineering-based Model and Online Content Assessment in Evaluating Ser-vice Design of Lazada Express. Proceedings of the 2020 The 6th International Conference on Industrial and Business Engineerin.

[46] McDougall S., Reppa I., Kulik J., Taylor A. (2016). What makes icons appealing? The role of processing fluency in predicting icon appeal in different task contexts. Appl. Ergon..

[47] Huang H., Yang M., Yang C., Lv T. (2019). User performance effects with graphical icons and training for elderly novice users: A case study on automatic teller machines. Appl. Ergon..

[48] Prasetyo Y.T., Castillo A.M., Salonga L.J., Sia J.A., Seneta J.A. (2020). Factors affecting perceived effectiveness of COVID-19 prevention measures among Filipinos during Enhanced Community Quarantine in Luzon, Philippines: Integrating Protection Motivation Theory and extended Theory of Planned Behavior. Int. J. Infect. Dis..

[49] Salman Y.B., Cheng H.-I., Patterson P.E. (2012). Icon and user interface design for emergency medical information systems: A case study. Int. J. Med. Inform..

[50] Lamy J.-B., Soualmia L.F., Kerdelhué G., Venot A., Duclos C. (2012). Validating the semantics of a medical iconic language using ontological reasoning. J. Biomed. Inform..

[51] Lindell M.K., Bostrom A., Goltz J.D., Prater C.S. (2021). Evaluating hazard awareness brochures: Assessing the textual, graphical, and numerical features of tsunami evacuation products. Int. J. Disaster Risk Reduct..

[52] Payre W., Diels C. (2019). Designing in-vehicle signs for connected vehicle features: Does appropriateness guarantee comprehension?. Appl. Ergon..

[53] Jung S., Park J., Park J., Choe M., Kim T., Choi M., Lee S. (2021). Effect of touch button interface on in-vehicle information systems usability. Int. J. Hum. Comput. Interac..

[54] Jylhä H., Hamari J. (2020). Development of measurement instrument for visual qualities of graphical user interface elements (VISQUAL): A test in the context of mobile game icons. User Model. User-Adapted Interact..

[55] Jylhä H., Hamari J. (2021). Demographic factors have little effect on aesthetic perceptions of icons: A study of mobile game icons. Internet Res..

[56] Cao Y., Zhang Y., Ding Y., Duffy V.G., Zhang X. (2021). Is an anthropomorphic app icon more attractive? Evidence from neu-roergonomomics. Appl. Ergon..

[57] Chen R., Huang J., Zhou J. (2020). Skeuomorphic or flat icons for an efficient visual search by younger and older adults?. Appl. Ergon..

[58] Chen M.-S., Lin M.-C., Wang C.-C., Chang C.A. (2009). Using HCA and TOPSIS approaches in personal digital assistant menu–icon interface design. Int. J. Ind. Ergon..

